# The Extract of *Crocus sativus* and Its Constituent Safranal, Affect Serum Levels of Endothelin and Total Protein in Sensitized Guinea Pigs

**Published:** 2013-09

**Authors:** Zahra Gholamnezhad, Hamed Koushyar, Goltaj Byrami, Mohammad Hossein Boskabady

**Affiliations:** 1 Applied Physiology Research Centre, and Department of Physiology, School of Medicine, Mashhad University of Medical Sciences, Mashhad, Iran

**Keywords:** Asthma, *Crocus sativus*, Endotheline, Inflammation, Safranal, Sensitization

## Abstract

***Objective(s):*** The effect of the extract of *Crocus sativus* and its constituent, safranal on inflammatory markers in sensitized guinea pigs was examined.

***Materials and Methods:*** Ovalbumin (OA) sensitized guinea pigs were given drinking water alone (group S), or drinking water containing three concentrations of safranal, three concentrations of extract and one concentration of dexamethasone, (n=6, for all groups) and serum levels of endotheline-1 (ET-1) and total protein (TP) were assessed.

***Results:*** Serum levels of ET-1 and TP in group S were significantly higher than control group (*P*<0.01 for ET-1 and *P*<0.001 TP). Treatment of S animals with dexamethasone, most concentrations of the extract and safranal significantly reduced serum levels of ET-1 and TP compared to group S (*P* <0.01 to *P* <0.001). The effects of one concentration of the extract and safranal were significantly higher than dexamethasone (*P* <0.05 to *P* <0.01).

***Conclusion: ***A preventive effect of the extract of *C. sativus* and its constituent safranal on serum inflammatory markers in sensitized guinea pigs was shown.

## Introduction

*Crocus sativus* L. is a small perennial plant which is mainly cultivated in Iran. Different constituents of the stigma of this plant are crocins, safranal, picrocrocin, ketoisophorone, isophorone, glycosidic terpenoids (1). The central part of the flower or female sexual organ (stigma) of *C. sativus* (Saffron) is used in traditional medicine for treatment of different diseases such as antispasmodic and expectorant (2).

The cytotoxic effect of saffron extract on HepG-2 and Hep-2 cell lines which is suggested to be due to a decrease in the NO concentration (3), its preventive effect on hematological parameters (4) and lung inflammation (5) of experimental asthmatic rats, its inhibitory effect on gastric cancer in rats (6) the effect of safranal on oxidative damage in rat hippocampus (7) and neuroprotective effects of crocin on reduction of free radicals-induced toxic effects (8) were shown recently. In addition, a relatively safe and normal profile for crocin in healthy volunteers at the given doses within the trial period was documented (9). Different uses such as a food additive and a palliative agent for many human diseases were described in a recent review (10). 

The relaxant effect of saffron on tracheal smooth muscle (11), inhibitory effect of the plant on histamine (H_1_) receptor (12) and its stimulatory effect on β-adrenoceptors (13) have been also shown. The effect of saffron on Th_1_/Th_2_ balance (14), its effect on total and differential WBC (15) as well as on pathological changes of the lung in sensitized animals (16) were also documented in our previous studies. Airway inflammation is the main characteristic future of asthma (17). Increased level of endothelin in bronchoalveolar fluid in asthmatics and its correlation with the severity of this disease was also observed (18).

With regard to anti inflammatory effect of *C. sativus* and its constituents, the effect of the extract of the plant and its constituent, safranal on endotheline-1 and total protein of sensitized guinea pigs was examined in the present study.

## Materials and Methods

***Plant extract and*** ***drugs***

*C. sativus *was provided by Novin Saffron which was collected from Ghaen, South Khorasan province (Middle East, Iran), and its stigma were dried at room temperature in the absence of sunlight. The plant was identified by Mrs. Molaei. A voucher specimen was preserved in the Herbarium of the School of Agriculture, Ferdowsi University of Mashhad (Herbarium No: 143-0319-1)*.* The hydro-ethanolic extract was prepared as follows: three grams of chopped *C. sativus* stigma were mixed with 50 ml ethanol 70% for 72 hr at room temperature and the solution was separated by maceration method. This process was repeated for three times. The solutions were dried in room temperature and stored in -4°C and away from light. Safranal was purchased from Fluka, Italy (Catalogue No.C4915, purity 75%).

*Animal groups and their sensitization*

The study was performed in control animals (group C) which were given drinking water alone and eight different groups of sensitized animals which were given drinking water alone (group S, an animal model of asthma) or drinking water containing the following agent during sensitization period (n=6 for each group):

1. 50 µg/ml dexamethasone (group S+D)
2. 0.1 mg/ml extract (group S+CS1)
3. 0.2 mg/ml extract (group S+CS2)
4. 0.4 mg/ml extract (group S+CS3)
5. 4 µg/ml safranal (group S+SA1)
6. 8 µg/ml safranal (group S+SA2)
7. 16 µg/ml safranal (group S+SA3)

Sensitization of animals to OA was performed using the method described previously (19). Briefly, adult Dunkin-Hartley guinea pigs (400-700 g, both sexes) were sensitized to OA (Sigma Chemical Ltd, UK) by IP injection of 10 mg OA and 100 mg Al (OH)_3_ dissolved in 1 ml saline on day one. One week later they were given 2 mg OA and 100 mg Al (OH)_3_ dissolved in 1 ml saline IP as a booster dose. From day 14 sensitized animals were exposed to an aerosol of 4% OA for 18±1 days, 5 min daily. The aerosol was administered in a closed chamber, dimensions 30 x 20 x 20 cm using a nebulizer (CX3, Omron Healthcare Europe B.V., Netherlands). Control animals were treated similarly but saline was used instead of OA solution. The study was approved by the Ethical Committee of Mashhad University of Medical Sciences. Animals were housed in individual cages with access to food and water *ad libitum* and were maintained at 22° ± 2°C on a 12 hr light/dark cycle (light period 0700 and 1900 hr).

**Figure 1:**
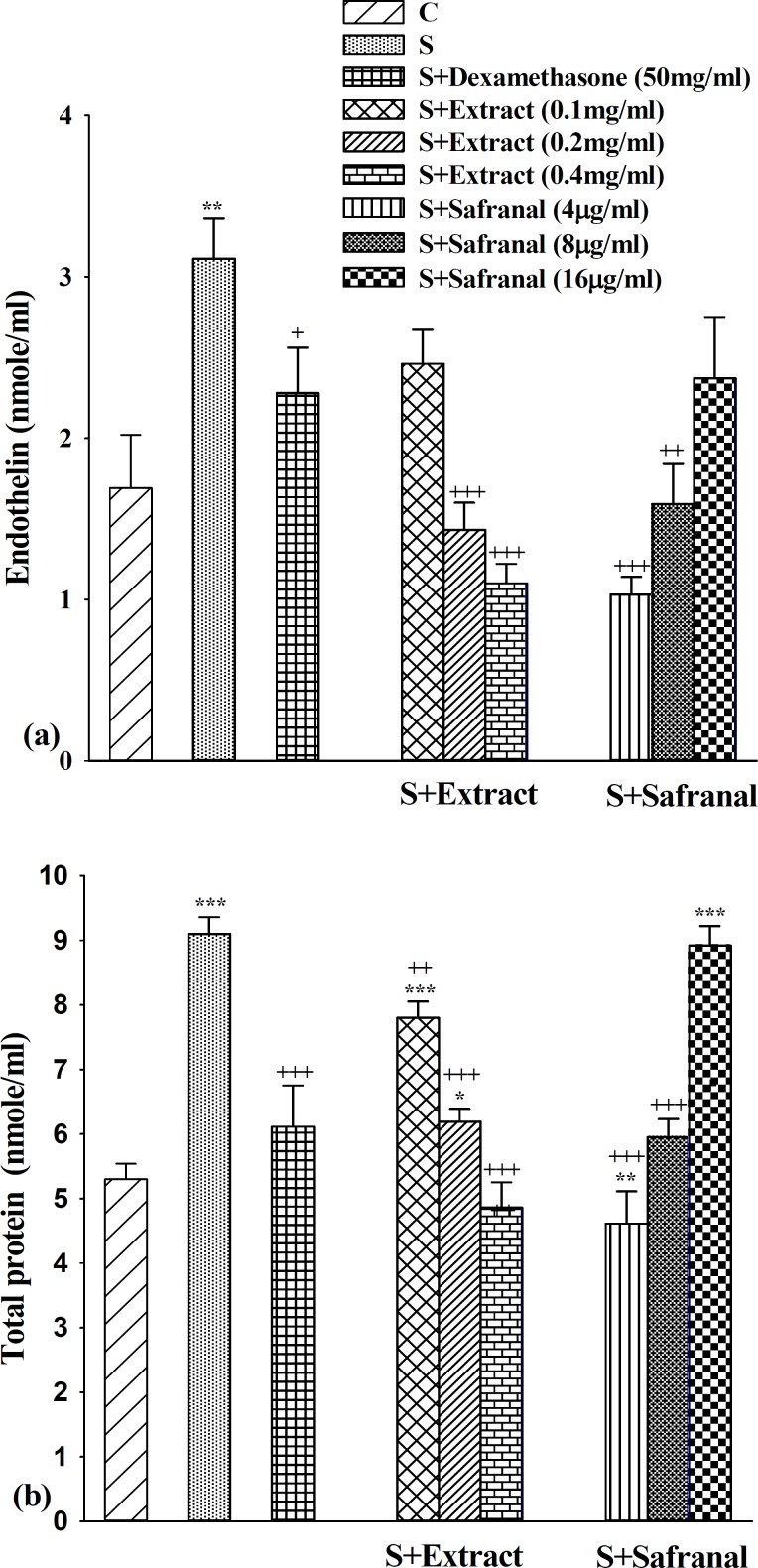
The levels of serum endothelin (a) and total protein (b) in control guinea pigs (C), sensitized animals (S), S treated with dexamethasone, three concentrations of the extract (S+Extract) and three concentrations of safranal (S+Safranal), (for each group, n=6). Statistical differences between control and different groups: *; *P*<0.05, **; *P*<0.01, ***; *P*<0.001. Statistical differences between treated *vs* sensitized group: +: *P*<0.05, ++: *P*<0.01, +++: *P* <0.001

***Measurement of serum endothelin-1 (ET-1)***

A total of 5 ml peripheral blood was obtained immediately after sacrificing the animals and placed at room temperature for 1 hr. The samples were then centrifuged at 3500×g at 4°C for 10 min. The supernatant was collected and immediately stored at 70°C until analysis. Serum endothelin was measured using the enzyme-linked immunosorbent assay (ELISA) Sandwich method according to the manufacturer’s instructions, (IBL’s ET-1 assay kit, Code No. 27165).

The protein contents of homogenates were determined using Bio-Rad protein assay kit (Bio- Rad Laboratories, US) according to the manufacturer protocol with photometric method. Briefly, a standard curve first using a standard protein was created. The sample is reacted with the dye in the same way, the absorbance measured, and the line equation from the standard curve is used to determine the concentration. 

***Statistical analysis***

All data were quoted as mean±SEM. According to the Kolmogorov Smirnov test these data had normal distribution. The comparison of data between groups were made using unpaired "t" test and unpaired one way ANOVA with Tuky Kermar *post hoc* test. Significance was accepted at *P* <0.05.

## Results

*Serum endothelin level*

Serum endothelin level in the group S was significantly higher than control group (*P* <0.01, Figure 1a). 

Treatment of sensitized animals with dexamethasone, two higher concentrations of the extract of *C. sativus* and two lower concentration of safranal lead to significant improvement in serum endothelin level (*P* <0.05 to *P* <0.001, Figure 1a). 

***Serum total protein level***

Serum total protein level in group S were significantly higher than that of group C (*P* <0.001, Figure 1b). In sensitized animal treated with all concentrations of the extract, two lower concentrations of safranal and dexamethasone, TP was significantly decreased (*P*<0.01 for low concentration of the extract and *P*< 0.001 for other cases, Figure 1b).

***Differences in in ET-1 and TP between dexamethasone, the extract and*** ***safranal***

The effects of low concentration of safranal and high concentration of the extract on both ET-1 and TP were significantly higher than the effect of dexamethasone (*P* <0.05 and *P* <0.01 for TP ant ET-1 respectively, Tables 1). The effect of medium concentration of the extract on ET-1 was also significantly higher than dexamethasone (*P* <0.05, Table 1). However, the effect of high concentration of safranal on serum TP level was significantly lower than dexamethasone (*P* <0.01, Table 1).

The effects of low concentration of safranal on both ET-1 and TP was significantly higher and its high concentration significantly lower than those of the extract (*P* <0.05 for high concentrations on ET-1 and *P* <0.001 for other cases, Tables 1).

***Differences in ET-1 and TP between three concentrations of the extract and*** ***safranal***

The effects of two higher concentrations of the extract on both ET-1 and TP were significantly higher than its low concentration (*P* <0.01 for medium and *P* <0.001 for high concentration in both ET-1 and TP, Tables 1). However, the effect of low concentration of safranal on both ET-1 and TP were greater than the effect of its high concentration (*P* <0.05 for ET-1 and *P* <0.001 for TP, Tables 1 and 2). Furthermore, the effect of high concentration of the safranal on total protein was lower than its medium concentration *P*<0.001, Table 1).

## Discussion

Anti-inflammatory effect of *C. sativus* and its constituents on different inflammatory conditions was shown previously. Therefore the preventive effect of extract from *C. sativus* and its constituent, safranal on inflammatory markers of sensitized guinea pigs was examined in the present study. The results showed increased serum ET-1 and TP levels in sensitized compared to control animal.

**Table 1:** Serum endothelin and total protein levels in control guinea pigs (C), sensitized animals (S), S treated with three concentrations (S+C1, S+C2 and S+C3) of the extract (CS), three concentrations of safranal (SA) and dexamethasone (S+D), (for each group, n=6)

Variable	Group	Control	S	S+D	S+C1	S+C2	S+C3
		1.69±0.33	3.11±0.25	2.28±0.28			
Endothelin	CS				2.46±0.21	1.43±0.17 ¶	1.10±0.12 ¶¶
	SA				1.03±0.11 ¶¶ ###	1.59±0.25	2.37±0.38 #
Total protein							
	CS	5.30±0.24	9.10±0.26	6.11±0.64	7.80±0.25	6.19±0.20	4.86±0.39 ¶
	SA				4.61±0.50 ¶ ###	5.95±0.28	8.92±0.30 ¶¶ ###

Values are presented as mean±SEM. Three concentrations of the extract were 0.1, 0.2 and 0.4 mg/ml and those of safranal were 4, 8 and 16 μg/ml and that of dexamethasone was 50 μg/ml.

Statistical *significance* for the *difference* between the data of S+D *vs.* treated groups with the extract and safranal: ¶; *P* <0.5, ¶¶; *P*<0.005.

Statistical *significance* for the *difference* between the data of safranal *vs.* extract: #; *P* <0.01, ###; *P* <0.001.

Statistical *significance* for the *difference* between the data of high and medium vs low concentrations of the extract or safranal: ; *P*<0.05, ; *P*<0.01, ; *P*<0.001

Statistical *significance* for the *difference* between the data of high vs medium concentrations of the extract or safranal: ; *P* <0.05,  ; *P* <0.001.

Treatment of sensitized animals with the extract and safranal prevented increased serum ET-1 and TP levels of sensitized guinea pigs. The effect of low concentration of safranal in improvement of serum ET-1 and TP levels in sensitized guinea pigs was higher but the effect of its high concentration was lower than the extract of *C. sativus*.

For treatment of asthma disease two types of drugs including bronchodilator for reliving bronchconstriction and prophylactic drugs for reducing airway inflammation, the main pathological feature of this disease. Therefore, the effect of the extract of *C. sativus*, and its constituent, safranal, on reduction of serum ET-1 and TP levels in sensitized guinea pigs suggest the suppressing effect of the plant and its constituent safranal on lung inflammation. Antioxidant effect of safranal (20) and anti-inflammatory and anti-oxidant effect of *C. sativus* (21) have been shown previously. The inhibitory effect of safranal on histamine (H_1_) receptor (12) and its antitussive effect (22) can also contribute to its anti-inflammatory effect. The results of all these studies could support the finding of the present study. 

The preventive effects of anti-inflammatory drugs on total WBC and eosinophil counts as well as histamine level (23) in sensitized and asthmatic patient were shown previously which support the anti-inflammatory effect of the extract of the plant and safranal proposed in the present study. Increased serum total protein was also shown in subjects with occupational asthma (24), which may be due to increased γ globulin, C reactive protein and other protein structured inflammatory mediators. Therefore, the reduction effect of the extract and safranal on TP of sensitized animal is the other evidence of preventive effect of the plant and its constituent on asthma. The comparable effects of the extract and safranal with dexamethasone is an other evidence indicating the anti-inflammatory effect of the extract of the plant and its constituent, safranal. Although the effect of dexamethasone was higher than the low concentration of the extract, its effect was lower than high concentration of the plant. In addition, the effect of low concentration of safranal was also higher than dexamethasone. Therefore, the preventive effect of the extract of *C. sativus* and safranal, one of the main constituent of the plant in sensitized guinea pigs were similar or even higher than the effect of dexamethasone.

The concentrations of the extract used in the present study were 0.1, 0.2 and 0.4 mg/ml and those of safranal were 4, 8 and 16 μg/ml. This means that the concentrations of the safranal were 0.04 times of those of the extract. In fact, the results showed that the lower concentration of safranal was more effective than its two higher concentrations on serum ET-1 and TP level in sensitized animals. These findings may indicate that the concentration of safranal close to that in the extract is more effective in sensitized animals. However, lower concentrations of safranal should be used in further studies to clarify this question. Therefore, the results of the present study may indicate that the preventive effect seen for the extract of the plant on sensitized guinea pigs is perhaps due to its constituent safranal. 

In ancient Iranian medical books, the therapeutic effects of this plant on respiratory diseases including asthma was indicated. A potent relaxant effect of the extract and safranal on tracheal chains, its stimulatory effect on ß_2_-adrnoceptors, inhibitory effect on histamine (H_1_) receptors, antitussive effect on guinea pigs, its effect on Th1/Th2 balance, the effect of the extract and safranal on pathological changes of the lung and serum histamine level as well as on total and differential WBC counts and tracheal responsiveness in sensitized guinea pigs were shown (11-16, 22, 25) previously. In the present study also the preventive effect of the extract and safranal on indices of lung inflammation in sensitized guinea pigs was shown. With regard to the results of all these studies, *C. sativus* and its constituent, safranal, could have a therapeutic effect on asthma both by bronchodilator and anti-inflammatory mechanisms.

## Conclusion

These results indicated the preventive effect of the extract of *C. sativus* and its constituent, safranal on serum ET-1 and TP levels in sensitized guinea pigs which could be indicated a prophylactic effect for the extract of the plant and safranal on asthma. The results also suggest that the effect of the plant is perhaps due to its constituent safranal. 
